# Successful Interruption of Transmission of *Onchocerca volvulus* in the Escuintla-Guatemala Focus, Guatemala

**DOI:** 10.1371/journal.pntd.0000404

**Published:** 2009-03-31

**Authors:** Rodrigo J. Gonzalez, Nancy Cruz-Ortiz, Nidia Rizzo, Jane Richards, Guillermo Zea-Flores, Alfredo Domínguez, Mauricio Sauerbrey, Eduardo Catú, Orlando Oliva, Frank O. Richards, Kim A. Lindblade

**Affiliations:** 1 Centro de Estudios en Salud, Universidad del Valle de Guatemala, Guatemala City, Guatemala; 2 Onchocerciasis Elimination Program for the Americas (OEPA), Guatemala City, Guatemala; 3 Ministerio de Salud Pública y Asistencia Social, Guatemala City, Guatemala; 4 Carter Center, Atlanta, Georgia, United States of America; 5 Division of Parasitic Diseases, National Center for Zoonotic, Vector-Borne and Enteric Diseases, Centers for Disease Control and Prevention, Atlanta, Georgia, United States of America; George Washington University, United States of America

## Abstract

**Background:**

Elimination of onchocerciasis (river blindness) through mass administration of ivermectin in the six countries in Latin America where it is endemic is considered feasible due to the relatively small size and geographic isolation of endemic foci. We evaluated whether transmission of onchocerciasis has been interrupted in the endemic focus of Escuintla-Guatemala in Guatemala, based on World Health Organization criteria for the certification of elimination of onchocerciasis.

**Methodology/Principal Findings:**

We conducted evaluations of ocular morbidity and past exposure to *Onchocerca volvulus* in the human population, while potential vectors (*Simulium ochraceum*) were captured and tested for *O. volvulus* DNA; all of the evaluations were carried out in potentially endemic communities (PEC; those with a history of actual or suspected transmission or those currently under semiannual mass treatment with ivermectin) within the focus. The prevalence of microfilariae in the anterior segment of the eye in 329 individuals (≥7 years old, resident in the PEC for at least 5 years) was 0% (one-sided 95% confidence interval [CI] 0–0.9%). The prevalence of antibodies to a recombinant *O. volvulus* antigen (Ov-16) in 6,432 school children (aged 6 to 12 years old) was 0% (one-sided 95% IC 0–0.05%). Out of a total of 14,099 *S. ochraceum* tested for *O. volvulus* DNA, none was positive (95% CI 0–0.01%). The seasonal transmission potential was, therefore, 0 infective stage larvae per person per season.

**Conclusions/Significance:**

Based on these evaluations, transmission of onchocerciasis in the Escuintla-Guatemala focus has been successfully interrupted. Although this is the second onchocerciasis focus in Latin America to have demonstrated interruption of transmission, it is the first focus with a well-documented history of intense transmission to have eliminated *O. volvulus*.

## Author Summary

Brought to the Americas from Africa by the slave trade, onchocerciasis is present in six countries in Latin America. The disease is caused by a round worm and is transmitted to humans by the bite of an infected black fly. Once in a human, the adult worms produce larvae that circulate through the body, causing itching or even blindness. Ivermectin, a drug that kills the larvae, is delivered by public health authorities in countries where the disease is present. If the larvae are killed, then the disease cannot be transmitted to more people. People living in the Escuintla-Guatemala focus, a region in Guatemala where the disease was common, have been taking ivermectin for many years. The Ministry of Health of Guatemala believes that onchocerciasis is no longer being transmitted in the area. To prove that there is no more transmission of the disease, the authors examined the eyes of residents of the area to see if they could find any evidence of the worms. They also conducted analyses of blood in school children to see if they had ever been exposed to the worm, and they caught thousands of black flies and tested them to see if they were infected. These evaluations found no evidence of transmission of the disease in the Escuintla-Guatemala focus. As a result, local public health authorities can stop giving ivermectin and invest their human resources in other important diseases.

## Introduction

Onchocerciasis (river blindness) is caused by a filarial nematode transmitted by black flies of the genus *Simulium* [1]. The disease may be mild (dermatitis) or severe (visual impairment and blindness) and is caused by the human immune response to microfilariae (mf) released by female adult worms as they move across subcutaneous tissue and spread throughout the body. Humans are the only known reservoir [2].

Onchocerciasis occurs throughout much of East and West Africa and Yemen, and was brought to the Americas through the slave trade [3]. It is now endemic to 6 countries in Latin America (Brazil, Colombia, Ecuador, Guatemala, Mexico and Venezuela). Foci of transmission in the Americas are relatively small and geographically delimited compared to areas of transmission in Africa [4]. In part due to the geographical isolation of foci, the goal of the Onchocerciasis Elimination Program of the Americas (OEPA) is both to eliminate ocular morbidity throughout the region, and to permanently interrupt transmission where possible [1],[5].

Control and eventual regional elimination of transmission is considered feasible due to the efficacy of ivermectin (Mectizan®, donated by Merck&Co, Inc.) as a microfilaricide, when used twice per year [6],[7]. While ivermectin used in this manner prevents transmission of infections, it does not kill adult worms [8] although it may reduce their fecundity and lifespan [9]. OEPA, along with its ministry of health counterparts, supports mass treatment with ivermectin twice per year, with the goal of reaching 85% of eligible individuals (those ≥5 years of age, ≥90 cm of height and ≥15 kg of weight; excluded are pregnant women and individuals with severe disease) living in endemic areas. Recent reanalysis of information on the effectiveness of ivermectin delivered in this strategy has suggested that six and a half years (13 treatment rounds) of such coverage can be sufficient to interrupt transmission [7].

**Figure 1:**
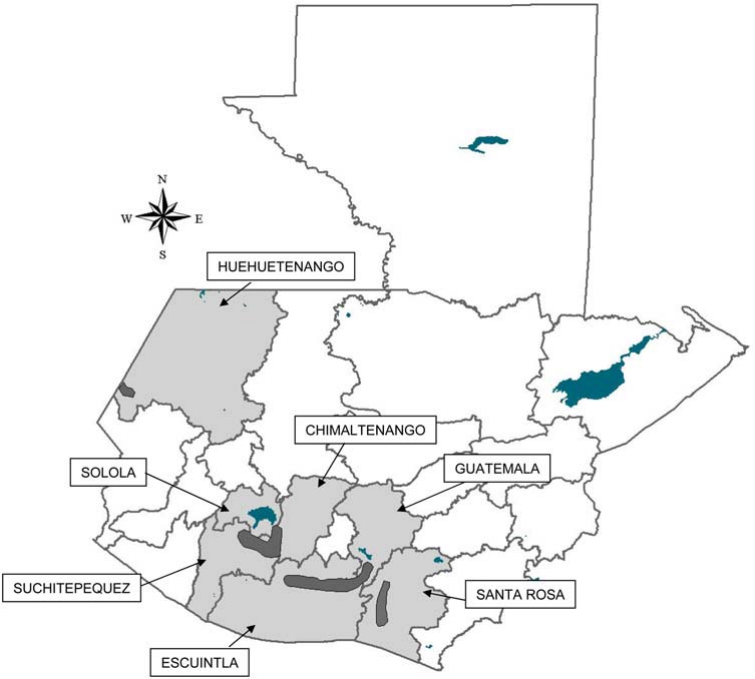
The four onchocerciasis foci in Guatemala (dark gray) in the endemic departments (light gray).

**Figure 2:**
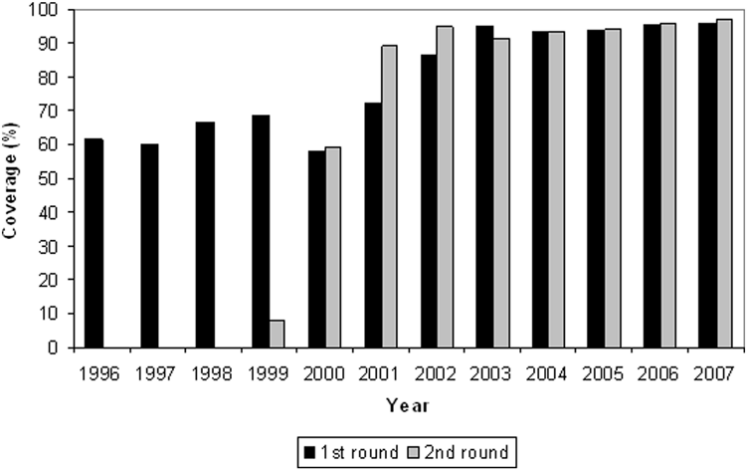
Ivermectin biannual treatment coverage from 1996 to 2007 in the Escuintla-Guatemala focus.

Guatemala, with a population eligible for treatment of 175,881 (to receive 351,762 treatments) in 2006 [5] accounts for 38.5% of the endemic population eligible for treatment in Latin America. Guatemala has four endemic foci: Santa Rosa (Department of Santa Rosa), Huehuetenango (Department of Huehuetenango), Escuintla-Guatemala (Departments of Escuintla and Guatemala) and the Central Endemic Zone (Departments of Suchitepéquez, Sololá and Chimaltenango; Figure 1) [10]. The Guatemalan Ministry of Public Health and Social Welfare (MSPAS, in its Spanish acronym) has been delivering ivermectin to endemic communities, through mass drug administration (MDA), since 1988 [8] and has reached 85% of the eligible population at risk twice per year in all foci since 2001 (Figure 2) [5].

Beginning in 2004, the MSPAS, in partnership with OEPA, the US Centers for Disease Control and Prevention (CDC) and the Universidad del Valle de Guatemala (UVG), began evaluating three of the four endemic foci in the country to determine whether transmission had been interrupted in these areas and if semiannual treatment could be suspended. Criteria for making these determinations are based on World Health Organization (WHO) guidelines presented in the 2001 document “Certification of Elimination of Human Onchocerciasis: Criteria and Procedures” [11] as adapted for field conditions by Lindblade *et al.* [12] and the OEPA steering committee (the Program Coordinating Committee-PCC) [1]. In summary, the criteria to be applied in areas with historically documented onchocerciasis transmission include: 1) demonstration of a prevalence of mf in the anterior segment (MfAS) of the eye (anterior chamber and cornea) to be less than 1%; 2) a cumulative incidence of *O. volvulus* infection of less than 0.1% in school-age children; and 3) a prevalence of infection in vectors of less than 0.05% [11],[13]. Based on these criteria, Lindblade *et al.* demonstrated that transmission had been interrupted in the Santa Rosa focus; [12] subsequently, the PCC recommended to the Minister of Public Health of Guatemala that treatment be suspended in this focus. That recommendation was accepted and the Santa Rosa focus is currently under a three year post treatment surveillance phase to monitor for transmission recrudescence [5].

In this report, we evaluate the current status of transmission of *O. volvulus* in the Escuintla-Guatemala focus based on the adapted WHO criteria.

## Methods

### The Escuintla-Guatemala focus

In 2007, the Escuintla-Guatemala focus consisted of 49,616 individuals at risk, with 45,224 eligible for treatment, divided among 103 communities in the department of Escuintla (14.30°N, 90.79°W), and 14 communities in the department of Guatemala (14.62°N, 90.53°W). Historically, this focus included areas with intense transmission: between 1979–1982, the community mf prevalence ranged from 8 to 38%. [14] A larval control effort from 1979–1989 significantly reduced both biting density and community mf prevalence.

### Potentially endemic communities (PEC)

**Figure 3:**
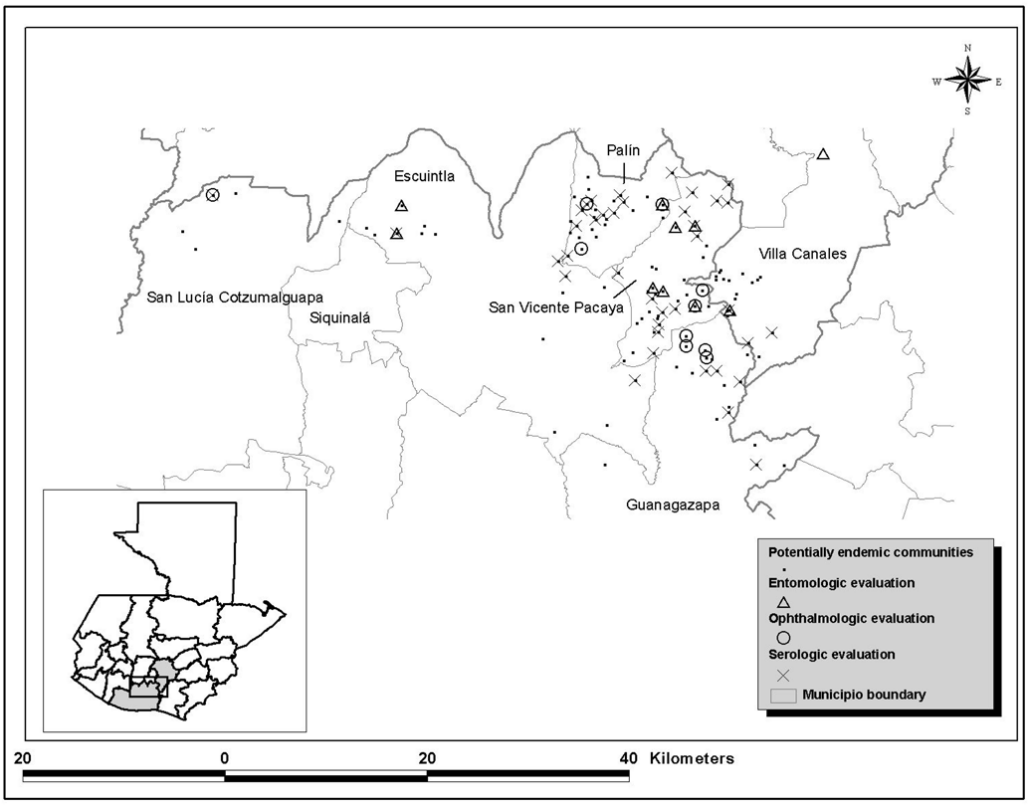
Potentially endemic communities and sites for the entomologic, ophthalmologic and serologic evaluations in the Escuintla-Guatemala focus.

To ensure that all areas with current or past evidence of onchocerciasis transmission were included in this evaluation, all communities with at least one of the following characteristics were identified using historical data, including unpublished reports from the MSPAS and published articles: a) past evidence of onchocerciasis transmission (nodules or mf in at least one community resident); b) suspicion of past transmission suggested by a documented survey, which may not have found evidence of transmission; or c) currently under semiannual ivermectin treatment by the MSPAS. A total of 155 communities satisfied at least one of these criteria) (Figure 3). These potentially endemic communities (PEC) served as the sampling frame for all evaluations of the status of transmission of onchocerciasis (Figure 3).

### Ophthalmologic evaluation

Because ocular morbidity is more likely to be found where onchocerciasis transmission is most intense, [15] we evaluated ocular lesions associated with onchocerciasis only in communities that historically had the highest rates of transmission. PEC with >0% nodule prevalence in the last 3 MSPAS surveys (1989, 1990 and 1991) and elevation >800 m were considered in order to maximize the possibility of finding *O. volvulus* related morbidity. A total of 16 communities satisfied these criteria. Two of these communities were dropped before the evaluation began because they had less than 5 inhabitants, and one was not included because it effectively serves as a bedroom community for Guatemala City, making it very difficult to locate potential participants in their homes.

The calculation of minimum sample size was based on estimating a population prevalence of MfAS of the eye of less than 1%. Finding 0 positive individuals out of 300 examined will allow a one-sided 95% confidence interval (CI) to exclude 1%. Given an estimated non-response rate of 10%, the total sample size required was 330.

All houses of the selected communities were mapped and the residents were censused using a pre-programmed hand-held personal digital assistant (PDA) with a global positioning system (GPS) attached. Eligible residents (those who were ≥7 years old and who had resided in the community for at least the last 5 years) were identified and recorded. As the ophthalmologist is capable of evaluating up to 90 individuals per day, we stratified communities into those with <90 eligible residents and those with ≥90 eligible residents. In the small communities (<90 residents), all eligible individuals (N∼266) were invited to participate. In the larger communities (≥90 residents), a PDA-based algorithm was applied in the field to randomly select 12% of the households and their members for inclusion in the evaluation (N∼223).

An ophthalmologist (OO) with extensive experience conducting evaluations of onchocerciasis-related eye disease performed the ophthalmologic evaluations. Visual acuity was measured with a Snellen chart using standard methods. Ocular examinations were conducted with a split-lamp in a darkened area after the patients were asked to sit with their head between their legs for 5 minutes [16]. MfAS were noted as live/coiled or dead/straightened. Data were entered in the PDA and later downloaded to a database for subsequent analysis.

### Serologic evaluation

We estimated the cumulative incidence of *O. volvulus* infection by measuring the prevalence of antibodies (IgG4) to a recombinant antigen of *O. volvulus*, OV-16, [17] in a stratified sample of school children 6–12 years old. We chose to stratify the sample into urban and rural schools because it was possible that levels of transmission would differ significantly between industrialized urban areas and rural communities located close to black fly breeding sites. The urban areas were taken to be the 2 large cities in the focus (San Vicente Pacaya and Palín), and the remainder of the schools located outside these cities were considered to be rural.

Information about schools and the number of children aged 6 through 12 who were attending was obtained with the help of the MSPAS and the Ministry of Education. Based on these figures, we estimated 4,674 eligible children in the urban schools and 9,815 in the rural schools. Schools were ordered at random within each stratum and then selected until the target sample size had been reached. The selected schools were visited and meetings were held with directors, teachers and parents to explain the evaluation. Teachers were asked to prepare a list of all enrolled children for the day of the evaluation.

Based on the WHO certification for elimination criteria (cumulative incidence <0.1%) and considering antibody prevalence equivalent to the cumulative incidence rate, 3,000 children were required in each stratum to calculate a one-sided 95% CI that excluded 0.1% when no seropositives were encountered. Given an expected 30% non-response rate, our target sample size was 4,286 in each stratum.

The methods used to collect finger-prick blood samples and data on residency from children participating in the evaluation have been described previously [12]. Briefly, each participant provided 80–120 uL of blood by standard sterile finger prick procedures. Whatman filter paper No. 2 was used to collect the blood directly after the finger prick. Children who didn't attend school on the appointed day were traced to their homes and asked to participate. Blood samples were processed within two months of collection using a standard ELISA [12].

### Entomologic evaluation

*Simulium* flies were collected from November 2005 to April 2006 (peak biting season) in seven PEC and tested for infectivity in order to calculate the Seasonal Transmission Potential (infected stage larvae per person per season, STP). Collection sites were selected through a rapid assessment of PEC to find those that satisfied the following criteria: a) high densities of *S. ochraceum*; b) presence of appropriate collection sites that capture areas where residents are most likely to be exposed to vectors (i.e. *casco*, near a house and *cafetal*, near coffee plantations); and c) willingness of the owner (in the case of *fincas* [plantations]) or residents to participate.

We used similar methods described by Lindblade *et al.* [12]. Two teams of two people (collector and paid attractant, a male resident of the *finca* ≥18 years old) rotated between two collection sites (*cafetal* and *casco*) in each PEC. The paid attractants were given ivermectin before starting collections and had finger-prick blood samples taken on filter paper at the beginning and at the end of the evaluation to evaluate exposure to *O. volvulus*. Collections started at 8:00 AM and ended at 5:00 PM taking 10 minute breaks at the end of every hour and a 1 hour break at noon. Each community was sampled two days per month.

At the laboratory at the UVG, the heads and thoraces of *S. ochraceum* were separated from their bodies and up to 50 flies were pooled per tube, maintaining separate months and communities. The bodies were analyzed first using a standard polymerase chain reaction (PCR) assay to detect *O. volvulus* DNA [18]. Positives were confirmed by a second PCR. If a positive was confirmed, all the fly heads of that community were tested.

### Statistical methods

The one-sided 95% confidence intervals for the prevalence of MfAS and antibodies to Ov16 were calculated using the SAS (version 9.0, SAS Institute, Cary NC) FREQ procedure with the EXACT statement, BINOMIAL option and an alpha level of 0.10. The Poolscreen 2.0 program was used to calculate the proportion of infective flies based on the number of positive pools [19]. Biting rates and STP were calculated according to standard methods [12].

### Ethics

All protocols received appropriate review and approval by the CDC (Atlanta, GA), the ethics committee of the UVG (Guatemala City, Guatemala), and the MSPAS (Guatemala City, Guatemala). All participants ≥18 years of age or the parents or guardians of children <18 years old read or had read to them an informed consent form and then were asked to sign or mark with their finger to indicate their consent to participate. Children aged 7 to <18 years old were read or had read to them an assent form and asked to sign or mark with their fingerprint to indicate their willingness to participate. Paid attractants also read or had read to them a consent form and indicated their willingness to participate with their signature or fingerprint.

## Results

### Ophthalmologic evaluation

Of the 13 communities selected for the evaluation, one could not be reached due to road and weather conditions, and the only family in a second community could not be found on the day of the evaluation. We evaluated 329 (73.1%) of the 450 eligible residents selected for inclusion in the evaluation. Of the total evaluated, 55% were women and 36% were 7–15 years old. Blindness due to onchocerciasis was not observed in any of the patients and 306 (93%) individuals evaluated had their visual acuity measured in the range of 20/20–20/70. No MfAS were found; the prevalence of MfAS was therefore 0, with a one-sided 95% CI of 0–0.9%.

### Serologic evaluation

In the urban area, we registered 4,674 enrolled children in 24 local schools, and 3,130 (67%) participated in the evaluation. Due to an insufficient blood sample, the results for seven children could not be determined. Out of the 3,123 samples analyzed, there was 1 positive (a 9 year old male living in an urban area) for antibodies against *O. volvulus*. A second blood sample was requested and also tested positive. The sample was sent for additional testing at an experienced onchocerciasis laboratory in Mexico (Instituto Politécnico Nacional, Reynosa, Mexico) against recombinant antigens Ov10, Ov11 and Ov16; the sample did not test positive for any of these antigens and we therefore concluded that it was a false positive and have recorded it as a negative result. In the rural area, we registered 4,614 enrolled children in 34 schools, and 3,316 (72%) participated in the evaluation. A total of seven samples again had to be discarded due to insufficient sample. None of the 3,309 samples tested were positive for OV-16. Therefore, the prevalence of antibodies to Ov16 in the Escuintla-Guatemala focus was 0, and the one sided 95% IC was 0–0.05%.

### Entomologic evaluation

A total of 28,423 *Simulium* flies were caught in 1,320 hours of sampling, from November 2006 through April 2007. None of the human attractants was found to be seropostitive for OV-16 either before or after the evaluation. Of the flies collected, 17,336 (61%) were *S. ochraceum* and 11,087 (39%) were *S. metallicum* (not considered to be a vector of onchocerciasis when community mf prevalence is low [20]). The highest biting densities were measured in November and December. A total of 14,099 *S. ochraceum* in 303 pools were tested for *O. volvulus* DNA by PCR; all pools were negative, thus, prevalence was 0% and the 95% CI was 0–0.01%. The geometric mean biting rate for *S. ochraceum* was 11.0 bites/person/hour while the arithmetic mean daily biting rate flies was 177 bites/person/day. As the proportion of infective flies was 0, the STP was also 0. To calculate the maximum potential STP, we used the upper end of the 95% CI of the proportion infective and the geometric mean biting rate to calculate the maximum STP, assuming that each infective fly would have 1 infective-stage larva. The calculated maximum potential STP was 1.0 infective stage larvae transmitted per person per season.

## Discussion

Our findings, based on the ophthalmologic, entomologic and serologic evaluations adapted from the WHO guidelines for certification of elimination, indicate that transmission of *O. volvulus* has been successfully interrupted in the Escuintla-Guatemala focus. Our studies in this formerly endemic area demonstrated that the prevalence of mf in the anterior segment of the eye was less than 1%, evidence of active or prior infection (or exposure) as measured by antibodies to a recombinant *O. volvulus* antigen (Ov-16) in school children was less than 0.1%, and *O. volvulus* DNA in vectors was under 0.05% (with a STP of 0 infective stage larvae per person per season).

**Figure 4:**
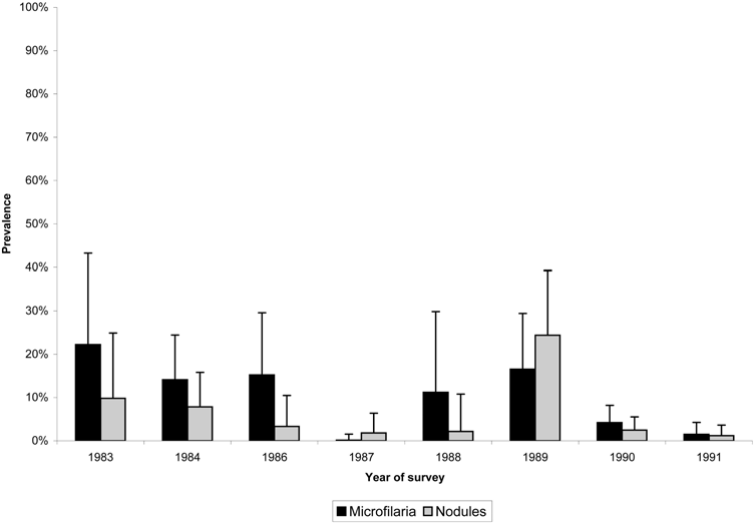
Mean community prevalence of microfilaria in the skin and nodules in the Escuintla-Guatemala focus. Unpublished Ministry of Public Health surveys from 1983 to 1991. Bars indicate the upper 95% confidence interval of the prevalence.

*O. volvulus* transmission in the Escuintla-Guatemala focus was extensively documented from 1979 to 1984 by the Guatemala-Japan Cooperative Project on Onchocerciasis Research and Control, which conducted a large-scale larval elimination program in the area around the town of San Vicente Pacaya in the Department of Escuintla [21]. Several communities in that area had a prevalence of mf in the skin of 8% to 38% as recently as 1982 [14]. Nevertheless, community mf prevalence dropped from an average of 26% to 7% during the years of the larval elimination program [14]. Larval control efforts ceased in 1989, and a MSPAS survey in1991 found community mf rates of 3% (MSPAS, unpublished data; Figure 4). No surveys had been conducted in Escuintla until the current report. However, the MSPAS provided semiannual ivermectin treatments in the focus, reaching more than 85% of the eligible population at risk twice per year from 2002 to 2007 (Figure 2).

Santa Rosa was the first focus in the Americas to demonstrate interruption of onchocerciasis transmission, but there is evidence that transmission was extremely low to nonexistent prior to ivermectin distribution [5]. In contrast, levels of transmission in the Escuintla-Guatemala focus were historically higher than the Santa Rosa focus and transmission was well documented until at least the early 1980s. The larviciding efforts in the San Vicente Pacaya area from 1983–1989 were responsible for a significant decline in vector biting rates and, subsequently, community mf prevalence. The successful interruption of transmission after 12–13 rounds of MDA with ivermectin in San Vicente Pacaya may be at least partially due to the reduction in mf prevalence resulting from the larviciding campaign. While other areas of the Escuintla-Guatemala focus experienced a decline in mf prevalence without vector control, larviciding may be considered a complementary strategy to mass drug administration in areas of intense transmission.

Although the reported specificity of the Ov-16 ELISA test is 90%, [17] our laboratory has now tested over 9,964 samples from endemic areas with only 1 false positive, a specificity of 99.99%. However, distinguishing false from true positives is challenging. We undertook extensive interviews of the family of the child initially found positive to rule out travel to other endemic areas or potential exposure to vectors during his daily activities. We tested other family members, including a grandfather who reported a nodule that was extirpated in the past, and none was found positive. After a second blood sample from the same child tested positive, we sent the samples for testing in another laboratory against additional antigens, where the child's samples were negative in all external tests. We, therefore, feel confident reporting this finding as a false positive.

The data presented in this report were extensively reviewed by OEPA and the PCC. Based on the results, the PCC recommended to the Minister of Health of Guatemala that ivermectin treatments be suspended in the Escuintla-Guatemala focus in 2008. The recommendation was accepted, and, as in Santa Rosa, three years of surveillance for recrudescence has now begun during which a final set of evaluations to ensure that transmission has been completely eliminated will be undertaken [5].

Currently we are conducting a similar series of evaluations in the focus of Huehuetenango along the border with Chiapas, Mexico, to determine whether transmission has been interrupted there. Results from these studies are expected mid-2008. As of the writing of this article, transmission of *O. volvulus* continues in the Central Endemic zone of Guatemala [5].

## Supporting Information

Alternative Language Abstract S1 Translation of the Abstract into Spanish by Rodrigo Gonzalez
(0.05 MB DOC)
