# Supplementary material for: Successful Interruption of Transmission of Onchocerca volvulus in the Escuintla-Guatemala Focus, Guatemala
Source: PLoS Negl Trop Dis. 2009 Mar 31;3(3):e404. doi: 10.1371/journal.pntd.0000404 (PMC2656640; doi:10.1371/journal.pntd.0000404)
Supplement: Alternative Language Abstract S1 — Translation of the Abstract into Spanish by Rodrigo Gonzalez (0.05 MB DOC) [file pntd.0000404.s001.doc]

Spanish Translation by Rodrigo Gonzalez

Fondo: La eliminación de la oncocercosis (ceguera de los ríos) en los 6 países de Latinoamérica en donde la enfermedad es endémica es considerada posible a través de la distribución en masa de ivermectina debido al tamaño relativamente pequeño de los focos y su aislamiento geográfico. En este estudio, evaluamos si la transmisión de oncocercosis ha sido interrumpida en el foco endémico Escuintla-Guatemala en Guatemala, basados en los criterios para la certificación de la eliminación de la oncocercocis de la Organización Mundial de la Salud (OMS).

Metodología/datos principales: Realizamos evaluaciones de morbilidad ocular y exposición a *Onchocerca volvulus* en humanos, al mismo tiempo capturamos al vector (*Simulium ochraceum*) para evaluar la presencia de ADN de *O. volvulus*. Todas las evaluaciones fueron realizadas en comunidades potencialmente endémicas (CPE, aquellas en donde hubiese o se sospechase historia de transmisión, o aquellas comunidades bajo tratamiento semianual con ivermectina) dentro del foco. La prevalencia de microfilarias en el segmento anterior del ojo en 329 individuos (≥7 años de edad, residentes de las CPE por al menos 5 años) fue 0% (intervalo de confianza de una cola de 95% [IC] 0-0.9%). La prevalencia de anticuerpos para un antígeno recombinante de *O. Volvulus* (Ov-16) en 6,432 niños en edad escolar (de 6 a 12 años de edad) fue 0% (IC de una cola del 95% 0-0.05%). De un total de 14,099 *S. ochraceum* evaluadas para determinar la presencia de ADN de *O. volvulus*, ninguna fue positiva (IC de una cola de 95% 0-0.01%). El potencial de transmisión por temporada fue, por lo tanto, 0 larvas en estado infectivo por persona, por temporada.

Conclusiones: En base a estas evaluaciones, la transmisión de oncocercosis en el foco Escuintla-Guatemala ha sido exitosamente interrumpida. A pesar de ser éste el segundo foco con oncocercosis en Latinoamérica que ha demostrado interrupción de transmisión, es el primer foco con historia bien documentada de transmisión intensa en el cual *O. volvulus* se ha eliminado.
